# Molten Salts Approach of Poly(vinyl alcohol)-Derived Bimetallic Nickel–Iron Sheets Supported on Porous Carbon Nanosheet as an Effective and Durable Electrocatalyst for Methanol Oxidation

**DOI:** 10.3390/gels9030238

**Published:** 2023-03-17

**Authors:** Badr M. Thamer, Meera Moydeen Abdul Hameed, Mohamed H. El-Newehy

**Affiliations:** 1 Department of Chemistry, College of Science, King Saud University, Riyadh 11451, Saudi Arabia; malhameed@ksu.edu.sa; 2 Department of Chemistry, Faculty of Science, Tanta University, Tanta 31527, Egypt

**Keywords:** PVA, bimetallic nickel–iron, molten salt synthesis, porous nanosheets, electrocatalyst, methanol oxidation

## Abstract

The preparation of metallic nanostructures supported on porous carbon materials that are facile, green, efficient, and low-cost is desirable to reduce the cost of electrocatalysts, as well as reduce environmental pollutants. In this study, a series of bimetallic nickel–iron sheets supported on porous carbon nanosheet (NiFe@PCNs) electrocatalysts were synthesized by molten salt synthesis without using any organic solvent or surfactant through controlled metal precursors. The as-prepared NiFe@PCNs were characterized by scanning and transmission electron microscopy (SEM and TEM), X-ray diffraction, and photoelectron spectroscopy (XRD and XPS). The TEM results indicated the growth of NiFe sheets on porous carbon nanosheets. The XRD analysis confirmed that the Ni_1−x_Fe_x_ alloy had a face-centered polycrystalline (fcc) structure with particle sizes ranging from 15.5 to 30.6 nm. The electrochemical tests showed that the catalytic activity and stability were highly dependent on the iron content. The electrocatalytic activity of catalysts for methanol oxidation demonstrated a nonlinear relationship with the iron ratio. The catalyst doped with 10% iron showed a higher activity compared to the pure nickel catalyst. The maximum current density of Ni_0.9_Fe_0.1_@PCNs (Ni/Fe ratio 9:1) was 190 mA/cm^2^ at 1.0 M of methanol. In addition to the high electroactivity, the Ni_0.9_Fe_0.1_@PCNs showed great improvement in stability over 1000 s at 0.5 V with a retained activity of 97%. This method can be used to prepare various bimetallic sheets supported on porous carbon nanosheet electrocatalysts.

## 1. Introduction

Over the past decades, great attention has been paid to develop high-performance clean energy devices as an alternative to fossil fuels, which cause many environmental problems. Fuel cells are one of high-performance alternative sources of energy that convert chemical energy directly into electrical energy without emitting a large amount of polluting gases [1,2]. One type of fuel cells, known as direct methanol fuel cells (DMFCs), operates at temperatures close to room temperature and uses methanol as fuel. DMFCs have been given special attention for development as they are candidates for use in many applications as automobiles, portable devices, and stationary power plants [3,4]. DMFCs rely on methanol as fuel, which is easy to store, refuel, and handle, and it has a high specific energy (up to 6 KWh/kg) [5,6]. Despite all these advantages, DMCFs still face some problems, such as the slow kinetics of oxidation of methanol fuel at the anode, methanol crossover, and cathode flooding, which affect the performance and conversion efficiency of DMFCs [7]. Therefore, many recent efforts have been focused on addressing all these problems to develop the performance of DMFCs. Despite the relative successes to overcome these issues, the slow kinetic of oxidation reaction of methanol at the anode requires further improvement for two reasons. First, the electrocatalysts used in the anode composition still rely on precious metals, which raises the cost of the DMFCs and, thus, limits their commercial use [8]. Second, the catalytic efficiency of catalysts based on noble metals such as platinum is still below the expected level due to the fact of their high susceptibility to poisoning by CO and HCO species [9,10]. So, there is a need to find alternative ways to overcome this problem and build anodes from nonprecious metals.

Among the most common metals, nickel-based catalysts are used as alternative catalysts in the anodes of DMFCs [11]. The catalytic activity of nickel-based catalyst is due to the formation of an effective NiOOH layer on the surface, which acts as an oxidizing agent for methanol oxidation [12,13]. Therefore, the catalytic activity of nickel catalysts depends on the thickness of the NiOOH layer, the formation of which is affected by the electronic structures and morphologies of nickel, as well as the concentration of the hydroxyl ion in the electrolyte [14]. Several studies have been conducted to optimize the morphology and chemical structures of nickel-based catalyst, which provide promising catalytic activity for the methanol oxidation [15,16,17]. Methanol is oxidized on the surface of nickel catalysts into carbon dioxide, water, and 6e^−^. However, the methanol oxidation reaction may not be completed and result in some intermediate compounds, such as formaldehyde and formic acid, depending on the type and composition of the catalyst [18]. The structures of nickel-based electrocatalysts have a significant impact on electrochemical properties, which is reflected in exposing the maximum active surface and facilitating electronic transfer [19]. One recent common strategy for increasing the efficiency of nickel-based catalysts is their downsizing. However, the small size of the nickel particles often agglomerates or grows during the electrocatalytic reaction, resulting in poor stability [20]. Moreover, the increase in thickness and crystallinity of the NiOOH layer on the surface weakens the conductivity of the nickel catalyst and hinders the charge transfer process [14]. Therefore, the appropriate strategy to overcome the aggregation problem is to fix nickel nanoparticles on a support material with a high surface area and conductivity such as carbon materials [21]. Porous carbon frameworks, carbon nanofibers, graphene, and carbon nanotubes have been used as supporting materials for nickel-based catalyst. For example, Thamer et al. found that the electrocatalytic activity of nickel nanoparticles supported on carbon nanofibers is higher than that of unsupported nanoparticles [22]. Another strategy to improve the catalytic activity and the stability of nickel-based catalyst for methanol oxidation is to combine them with other transition metals [23,24]. So far, a number of methods have been reported in the preparation of carbon material-supported bimetallic nanostructures, such as electrodeposition [25], solvothermal method [26], and pyrolysis [27], but some of these methods fail to control the size of the metal nanoparticles or require complex and harsh conditions. Molten salt synthesis is a facile, efficient, clean, and low-cost method for preparing metallic catalysts supported on porous carbon materials [28,29,30,31,32,33,34]. The molten salts are readily available, low in cost, nontoxic, reusable, and thermally stable during the calcination process, serving as a template for the creation of porous materials. Furthermore, various salts such as NaCl, NaCl−KCl, ZnCl_2_, ZnCl_2_-NaCl, and KCl/LiCl can be used to synthesize mono/bimetallic catalysts supported on porous carbon. Compared with other methods, whether wet or solid synthesis route, the molten salt method has many unique advantages. For example, the molten salt liquid medium facilitates the flow of solids through the features of convection and diffusion, lowering the heat of product production. Moreover, molten salt has a high ability to solvate many metal ions and inorganic materials, and it has a high solubility in aqueous media, which allows it to be separated from the product and reused [34,35,36,37].

Bimetallic nickel–iron alloys have a numerous applications, including as electrocatalyst for oxygen evolution reactions [38], water splitting [39], and rechargeable zinc–air batteries [40]. The preparation of effective electrocatalysts based on bimetallic nanoparticles supported on carbon nanosheets without the need for surfactants and other reagents is still a challenge. Moreover, the use of bimetallic nickel–iron alloys for methanol oxidation is scarce, and the preparation of nickel–iron sheets supported on porous carbon nanosheets by molten salt synthesis remains elusive. Herein, we demonstrate the synthesis of bimetallic nickel–iron alloy supported on porous carbon nanosheets (NiFe@PCNs) by a single-step molten salt without the use of capping agents or organic solvents. As templates, mixtures of potassium chloride and lithium chloride (KCl/LiCl) eutectic molten salts and poly(vinyl alcohol) (PVA) as a precursor of porous carbon were used. Moreover, nickel acetate and iron acetate were used as precursors. KCl/LiCl eutectic salt can act as a solvent at a temperature above 335 °C to form nickel–iron and porous carbon nanosheet substrates. Such Ni_0.9_Fe_0.1_@PCN catalysts showed interesting electrocatalytic activity and the stability for methanol oxidation compared to Ni@PCs and other alloys. Furthermore, this method offers a large-scale route for the synthesis of bimetallic electrocatalysts supported on porous carbon.

## 2. Results and Discussion

### 2.1. Structure and Morphology of the Catalysts

**Figure 1:**
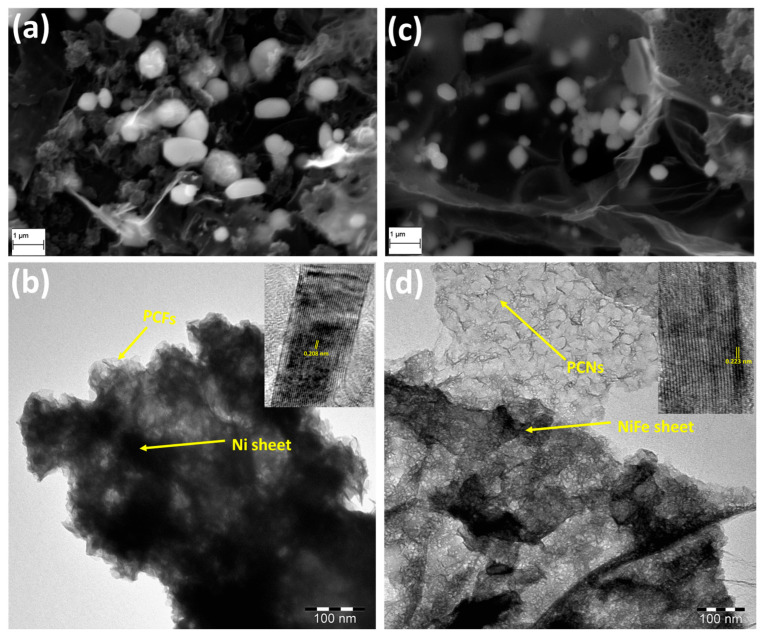
SEM and TEM images of the (**a**,**b**) Ni@PCFs and (**c**,**d**) Ni_0.9_Fe_0.1_@PCNs.

In order to obtain the morphology of the NiFe@PCN nanosheets, SEM and TEM microscopic characterizations were performed. As displayed in the SEM images (Figure 1a,c), the morphology of Ni@PCFs and NiFe@PCNs clearly shows curled, randomly aggregated, and crumpled porous sheets that are decorated with metal particles that have a cubic crystal structure. Furthermore, TEM was used to study the morphology of the catalyst with and without doping by iron, and the findings are displayed in Figure 1b,d. A TEM image of the Ni@PCFs shows a condensed sheet-like shape supported on a porous carbon structure (Figure 1b). In contrast, a TEM image shows that doping the catalyst with iron resulted in thinner metallic sheets supported on highly porous carbon nanosheets, as displayed in Figure 1d. Carbon nanosheets have mixed micro/mesoporous structures, which allow for a higher rate of electrolyte diffusion within the catalyst structure. A representative HRTEM image (inserts in Figure 1b,d) displays that Ni@PCF and NiFe@PCN samples have lattice fringes with values of 0.208 and 0.223 nm for the (111) plane of nickel and nickel–iron phase, respectively, showing their crystalline structure.

**Figure 2:**
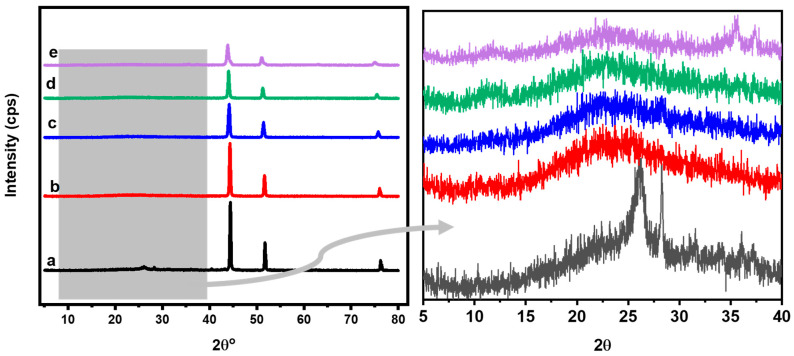
XRD of the (a) Ni@PCs; (b) Ni_0.9_Fe_0.1_@PCNs; (c) Ni_0.8_Fe_0.2_@PCNs; (d) Ni_0.7_Fe_0.3_@PCNs; (e) Ni_0.60_Fe_0.4_@PCNs.

The crystalline structures and the particle size of the catalyst were investigated by XRD, as displayed in Figure 2. The catalyst without iron (Ni@PCs) exhibits three sharp peaks at 44.4°, 51.76°, and 76.31° that match to the face-centered cubic (fcc) structure of the Ni planes, (111), (200), and (220), respectively [41,42]. For the Ni-Fe bimetallic, the XRD patterns show peaks similar to those of the Ni@PCs with a shift of the peaks towards lower *2θ* values, decreasing in their intensity and increasing their width with an increasing iron ratio, indicating a marked change in the phase and crystallinity with the incorporation of iron. The crystallite sizes of the Ni-Fe were analyzed using Scherrer’s equation, and the results show that their sizes decreased with an increasing iron content, as shown in Table 1. The obtained result is consistent with the result obtained by Xiang et al., who used a different method to prepare Ni-Fe nanoparticles [43]. The average metal crystallite sizes of the electrocatalyst were calculated to be 30.59 nm (Ni@PCs), 27.73 nm (Ni_0.8_Fe_0.2_@PCNs), 21.69 nm (Ni_0.8_Fe_0.2_@PCNs), 23.55 nm (Ni_0.7_Fe_0.3_@PCNs), and 15.51 nm (Ni_0.6_Fe_0.4_@PCNs). It is noteworthy that a broad peak in the XRD spectra of all samples appeared in the 23.65° to 26° range and corresponds to (002) carbon facets, indicating the presence of porous carbon nanosheets. It was noticed that the d-space increased with increasing the iron ratio in the catalyst, which could intercalate into the graphite layers and increase the exposure of the active sites. These values result in an interlayer spacing (d_002_) of 0.3543–0.3597 nm for the NiFe@PCNs samples and 0.3417 nm for the Ni@PC samples. Based on the well-known Bragg equation, the crystalline size along the c-axis in the graphitic lattice can be estimated to be 0.57–0.78 nm for the NiFe@PCNs samples and 8.0 nm for the Ni@PCs. Therefore, the CNs were composed of approximately two layer-stacked sheets (e.g., 0.781/0.3758 = 2) for the NiFe@PCNs samples and eight layers for the Ni@PCs, as shown in Table 1. The porous structure and less layer-stacked sheets in the NiFe@PCN samples correspond to good electron transfer and electrolyte diffusion, which are advantageous in electrochemical applications.

**Figure 3:**
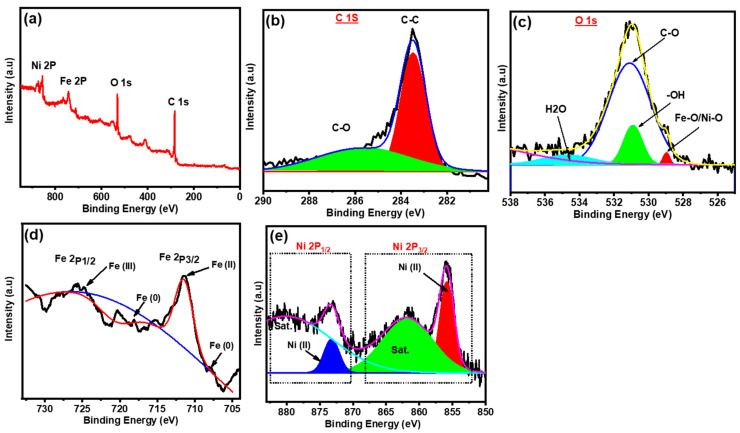
XPS analysis (**a**) survey scan spectrum of Ni_0.9_Fe_0.1_@PCNs and (**b**–**e**) high-resolution fitting of (**b**) C 1s, (**c**) O 1s, (**d**) Fe 2p, and (**e**) Ni 2p.

The chemical composition/electronic structure of the catalyst was investigated using XPS measurements. As displayed in (Figure 3a), XPS spectrum reveals the presence of carbon, oxygen, iron, and nickel in the NiFe@PCN sample. High-resolution scan of C 1s (Figure 3b) displays two bands appear, one of which is strong at 283.5 eV and the other weak at 286 eV, and they are related to the *sp*^2^ C-C bond and the C-O bond, respectively. The high-resolution scan of O 1s (Figure 3c) shows a weak band at 529 eV, which is attributed to Ni-O/Fe-O composition, while the strong band located at 531 eV is attributed to C-O bond [44]. The high-resolution scan of Fe 2p (Figure 3d) can be divided into three pair doublets at 708/718.12 eV, 709/711.2 eV, and 712.6/725.65 eV, which belong to Fe (zero-valent), Fe^2+^ (Fe 2p_3/2_/Fe 2p_1/2_), and Fe^3+^ (Fe 2p^3/2^/Fe 2p_1/2_), respectively [45]. High-resolution scan of Ni 2p (Figure 3e) displays four primary peaks for Ni element composition. Ni^2+^ (e.g., NiO and Ni(OH)_2_) has two conventional peaks at 856 and 873.3 eV, whereas the satellite peaks have two additional peaks at 862 and 880.3 eV [46].

**Figure 4:**
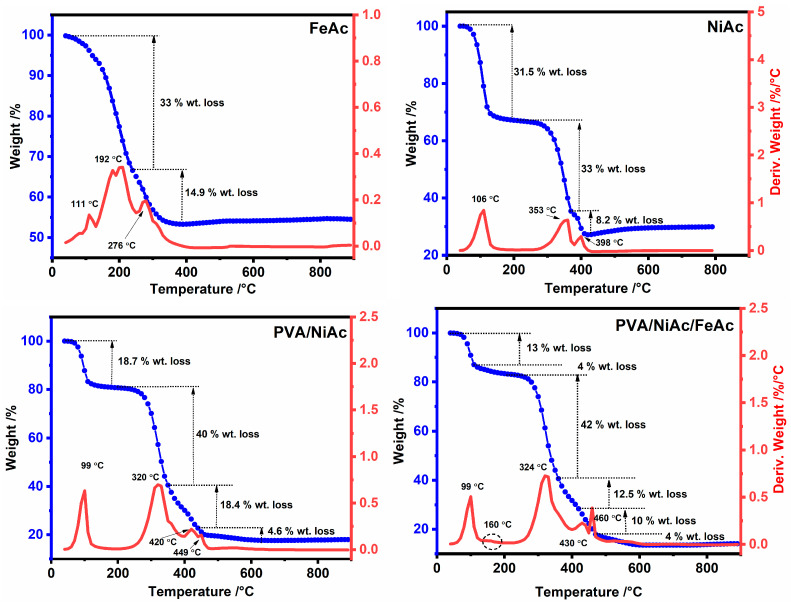
TGA/DTA of FeAc, NiAc, PVA/NiAc, and PVA/NiAc/FeAc.

To test the thermal decomposition behavior of the used precursors for the preparation of the catalyst and to regulate the optimal conditions for calcination, thermogravimetric analysis (TGA/DTA) was done. Figure 4 displays the thermal decomposition study of FeAc, NiAc, NiAc/PVA, and FeAc/NiAc/PVA mixtures with the temperature changing from 30 to 800 °C under N_2_. The TGA/DTA curve for FeAc shows that the thermal decomposition took place in two steps. The first one was carried out in range of 100—240 °C, with weight loss of 33%. This corresponds to the theoretical value of the acetone molecule loss and the formation of ferric carbonate. In the second step, the weight loss was 14.9%, which can be assigned to the decomposition of ferric carbonate to ferrous oxide and the release of carbon dioxide. However, the residual weight at 400 °C was 53.5%, which is higher than the theoretical weight of ferrous oxide (46%), indicating a mixture of iron oxide and iron carbide formation [47]. The thermal decomposition of NiAc is shown to have taken place in three main steps. The weight loss in the first step was 31.5% and took place in the thermal range between 50 and 140 °C. That weight loss theoretically corresponds to the weight ratio of four water molecules in nickel acetate, indicating the liberation of the physically bound water molecules and is called the dehydration process. It is noted in the second step that it was made at a temperature between 280 and 380 °C, and the weight lost was approximately 33%. This is due to the decomposition of nickel acetate anhydrous into nickel carbonate through the loss of an acetone molecule, where the weight loss matches pretty well with the theoretical weight of acetone. At temperatures above 380 °C, nickel carbonate decomposed to nickel oxide, which is then partially reduced by carbon monoxide into nickel [48]. At 398 °C, the residual weight was 27%, while the calculated weight percentage of nickel and nickel oxide is 23.4% and 29.8%, respectively that approved the formation of a mixture of nickel and nickel oxide. The TGA/DTA analysis of the NiAc/PVA (weight ratio 1:1) was also carried out to understand the thermal decomposition behavior of the mixture. It is known that PVA is thermally stable at a temperature less than 200 °C [49] and, therefore, the first step is attributed to the dehydration process of NiAc, which took place between 70 and 120 °C with weight loss of 18.7%. The second step took place in the range between 260 and 350 °C with weight loss of 40%, which is higher than the weight lost in the second step for the thermal decomposition of pure NiAc. This is assigned to the simultaneous thermal decomposition of PVA and NiAc in the second step, as well as in third step. The thermal decomposition of the mixture shows the presence of fourth step above 440 °C, which is probably assigned to the reduction of Ni(II) to Ni (0) by carbon monoxide. The thermal decomposition of NiAc/FeAc/PVA at the weight ratio 1:0.05:1 was also studied by TGA/DTA analysis and takes place in several steps. The first step (at 100 °C) is assigned to the dehydration of NiAc, while the second (at 160 °C) is attributed to the decomposition of FeAc. The third step is attributed to the simultaneous thermal decomposition of the NiAc, FeAc into carbonate, and the breakdown of the PVA backbone. The weight lost in the third step is the highest (42%) and is attributed to the simultaneous thermal decomposition of NiAc and FeAc to carbonates, as well as the degradation of PVA backbone. The two steps that took place at temperatures between 360 and 480 °C are assigned to the thermal decomposition of the intermediate compounds produced in the third step and their reduction to nickel and iron. The final step at a temperature above 550 °C is attributed to the graphitization process [50]. To summarize, the optimal conditions for calcination of the NiAc/FeAc/PVA mixture are temperatures above 600 °C in a nitrogen atmosphere.

### 2.2. Electrocatalytic Activity and Durability of Catalysts

The utilization of nonprecious electrocatalysts (e.g., nickel-based catalysts) as an alternative to precious platinum-, ruthenium-, and palladium-based catalysts is one way to reduce the cost of methanol fuel cells. However, the sluggish methanol oxidation process onto the surface of the nickel-based catalyst significantly hampers its use as an alternative to precious metal-based catalysts.

**Figure 5:**
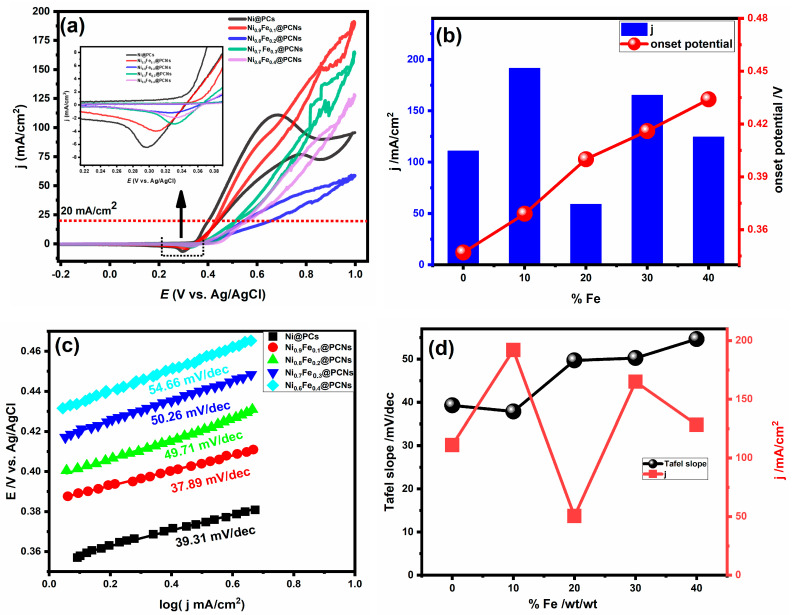
(**a**) CV of Ni@PCs, Ni_0.9_Fe_0.1_@PCNs, Ni_0.8_Fe_0.2_@PCNs, Ni_0.7_Fe_0.3_@PCNs, and Ni_0.60_Fe_0.4_@PCNs in 1.0 M methanol/1.0 M KOH at 0.5 V/s; (**b**) plot % Fe ratio vs. current density and onset potential; (**c**) Tafel plots of Ni@PC, Ni_0.9_Fe_0.1_@PCN, Ni_0.8_Fe_0.2_@PCN, Ni_0.7_Fe_0.3_@PCN, and Ni_0.60_Fe_0.4_@PCN electrodes in 2M methanol/1M KOH; (**d**) plot % Fe vs. Tafel slope value and current density.

**Figure 6:**
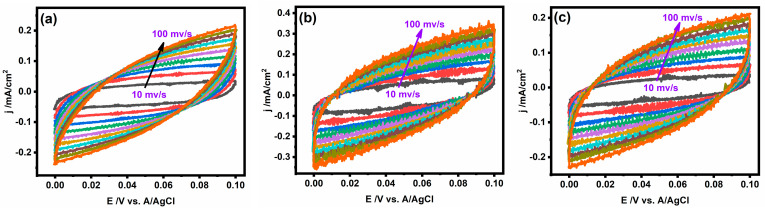

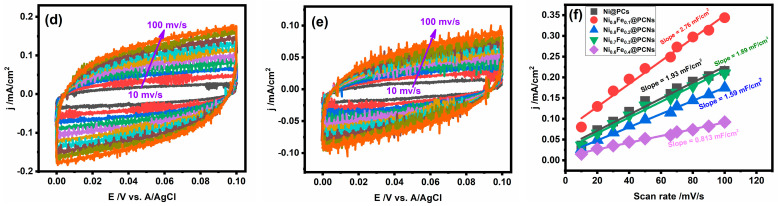
CV of (**a**) Ni@PCs; (**b**) Ni_0.9_Fe_0.1_@PCNs; (**c**) Ni_0.8_Fe_0.2_@PCNs; (**d**) Ni_0.7_Fe_0.3_@PCNs; (**e**) Ni_0.60_Fe_0.4_@PCNs measured in a nonFaradaic region at different scan rates in 1 M KOH; (**f**) linear fit of anodic charging current density measured at 0.1 V vs. Ag/AgCl vs. scan rate.

Among the various methods, the method of incorporation of a co-metal with nickel catalysts has received intense research interest due to the synergistic effect. Therefore, the effect of the incorporated iron ratio on the electrocatalytic activity of the Ni@PC catalyst was studied using cyclic voltammetry in 1.0 M of methanol and KOH at a scanning rate of 50 mV/s, as shown in Figure 5a. The incorporated iron ratio played a significant role in the current density of methanol oxidation, but the electrocatalytic activity of the catalyst for methanol oxidation demonstrates a nonlinear relationship with the iron ratio. The maximum current densities of Ni@PCs, Ni_0.9_Fe_0.1_@PCNs, Ni_0.8_Fe_0.2_@PCNs, Ni_0.7_Fe_0.3_@PCNs, and Ni_0.6_Fe_0.4_@PCNs were 110.9, 191.34, 59.04, 165.13, and 124.44 mA/cm^2^, respectively. The outstanding performance of the Ni_0.9_Fe_0.1_@PCNs for methanol oxidation can be assigned to the synergistic effect between the formation of nickel and the incorporation of iron. The NiOOH layer, which is formed at a low potential, is the active layer for methanol oxidation and doping nickel through an iron-enhanced electrocatalytic activity, because doping changes the electronic structure of the catalyst. However, doping nickel with more than 10% iron resulted in decreasing the electrocatalytic activity due to the inability of NiOOH layer to form, as displayed in Figure 5a. To evaluate the effect of the incorporated iron on the methanol oxidation mechanism on the surface of the catalyst, Tafel slope analysis was performed. Figure 5c shows the Tafel slopes of the Ni@PCs, Ni_0.9_Fe_0.1_@PCNs, Ni_0.8_Fe_0.2_@PCNs, Ni_0.7_Fe_0.3_@PCNs, and Ni_0.60_Fe_0.4_@PCNs obtained by plotting the logarithm of current density versus onset potential. The values of Tafel slopes obtained for the as-prepared catalysts take the following order: Ni_0.9_Fe_0.1_@PCNs (37.89 mv/dec) < Ni@PCs (39.31 mV/dec) < Ni_0.8_Fe_0.2_@PCNs (49.71 mV/dec) < Ni_0.7_Fe_0.3_@PCNs (50.26 mV/dec) < Ni_0.60_Fe_0.4_@PCNs (54.66 mV/dec). The results revealed that the incorporation of iron by 10% resulted in a decrease in the Tafel slope value compared to the absence of iron (Ni@PCs), whereas higher percentages of iron resulted in a significant increase in the Tafel slope values. This result indicates that the kinetics of methanol oxidation at the surface of the Ni_0.9_Fe_0.1_@PCN electrode were the fastest. This result also shows that, while the Ni_0.9_Fe_0.1_@PCN electrode was the most electroactive toward methanol oxidation of all tested electrodes, its onset potential was higher than Ni@PCNs. Iron incorporation within Ni@PCs causes a shift in the onset potential to higher values, as well as a decrease in the redox peak (shown in the insert of Figure 5a), which is consistent with previous reported studies [51]. The decrease in the redox peaks and shift to a high onset potential can be assigned to iron suppressing the formation of NiOOH layer [52]. The electrocatalytic activity is consistent with the estimated electrochemically active surface area (ECSA) of the prepared electrocatalysts, as displayed in Figure 6. The ECSA of the prepared catalysts was calculated by estimating the system’s double-layer capacitance using CV measurements. The ECSA values were 48.25, 69.0, 39.75, 47.25, and 20.32 cm^2^ for the Ni@PCs, Ni_0.9_Fe_0.1_@PCNs, Ni_0.8_Fe_0.2_@PCNs, Ni_0.7_Fe_0.3_@PCNs, and Ni_0.60_Fe_0.4_@PCNs, respectively.

**Figure 7:**
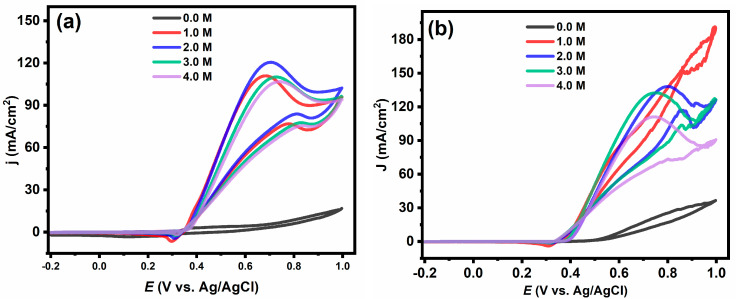

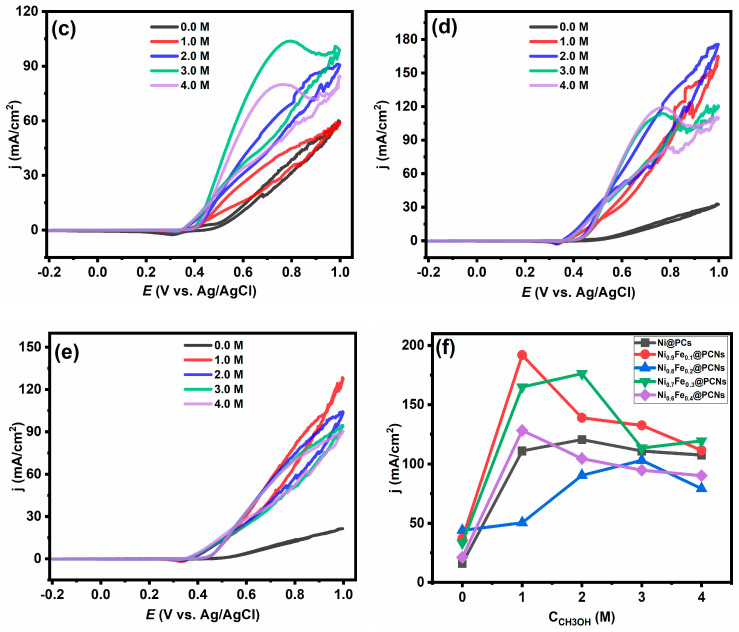
Effect of methanol concentration on the electrocatalytic oxidation of methanol on (**a**) Ni@PC; (**b**) Ni_0.9_Fe_0.1_@PCN; (**c**) Ni_0.8_Fe_0.2_@PCN; (**d**) Ni_0.7_Fe_0.3_@PCN; (**e**) Ni_0.6_Fe_0.4_@PCN electrodes in 1.0 M KOH solution at 50 mV s^−1^; (**f**) plot of the methanol concentration vs. current density for all electrodes.

The electrochemical performance of the catalyst was greatly affected by changing the methanol concentration in the electrolyte. As shown in Figure 7a–e, the addition of 1 M of methanol led to a higher current density compared to its absence (only 1 M of KOH) for all prepared catalysts. Moreover, the obtained results indicated that the increase in the current density was not linear with increasing the methanol concentration and that the optimal concentration of methanol varies with the iron ratio. The optimal concentration of methanol was 1.0 M for Ni_0.6_Fe_0.4_@PCNs and Ni_0.9_Fe_0.1_@PCNs, 2.0 M for Ni@PCs and Ni_0.7_Fe_0.3_@PCNs, and 3.0 M for Ni_0.8_Fe_0.2_@PCNs. The difference in the optimal concentrations of methanol refers to the diverse number of the active sites on the catalyst surface. The decrease in the current density at high methanol concentrations is attributed to methanol and intermediates coating the surface of the catalyst, which prevents hydroxyl ions from reaching the active sites on the surface [53]. Furthermore, the increase in methanol concentration was accompanied by increase in electrolyte resistance, which results in a decrease in the current density [54]. An interesting result is that the current density of the Ni@PC catalyst decreased after a 0.7 V at different concentrations of methanol. This result indicates the poisoning of the noniron doped catalyst at high potential. On the other hand, it was found that the Ni_0.9_Fe_0.1_@PCN, Ni_0.8_Fe_0.2_@PCN, and Ni_0.7_Fe_0.3_@PCN catalysts resisted poisoning at concentrations of 1.0 and 2.0 M, while the Ni_0.6_Fe_0.4_@PCN catalyst resisted poisoning at all concentrations under study despite the low current density.

**Figure 8:**
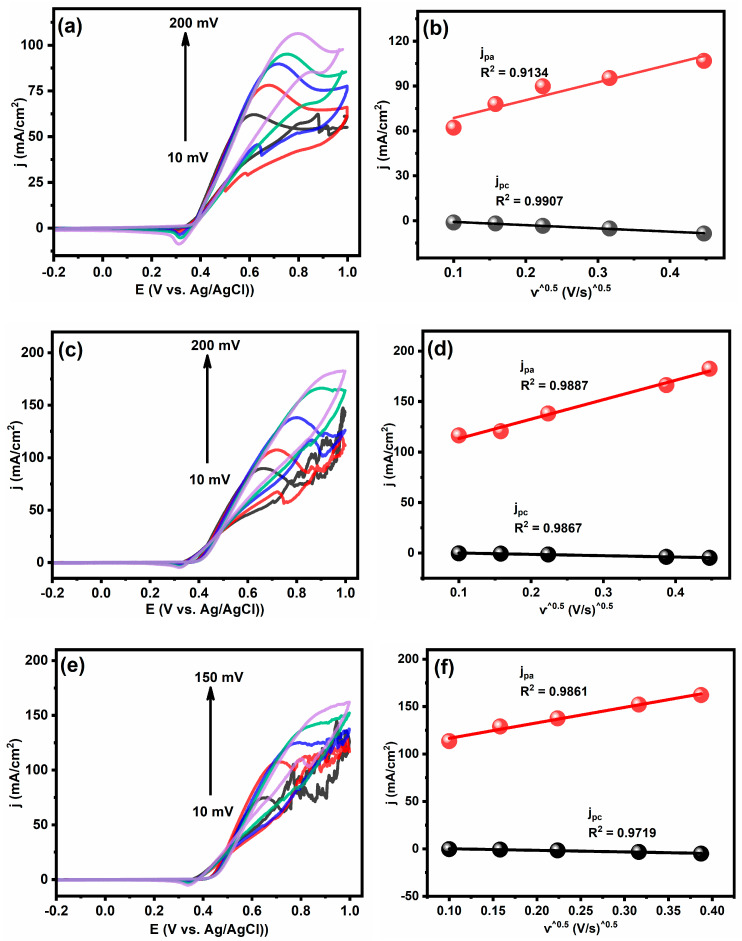

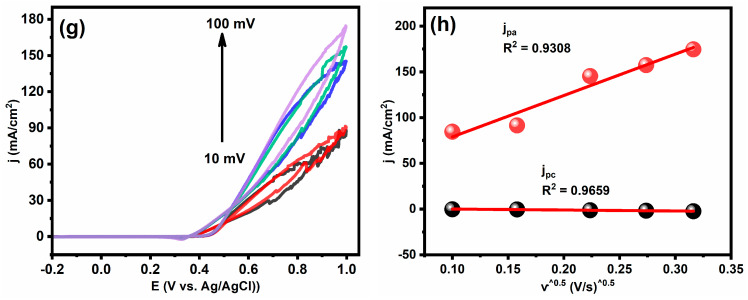
Effects of the scan rate on the electrocatalytic oxidation of methanol and plots of the square root of the scan rate vs. cathodic and anodic current density for (**a**,**b**) Ni@PCs; (**c**,**d**) Ni_0.9_Fe_0.1_@PCNs; (**e**,**f**) Ni_0.7_Fe_0.3_@PCNs; (**g**,**h**) Ni_0.6_Fe_0.4_@PCNs.

The effect of the scan rate on the peak of current density for fabricated electrodes in 1 M of CH_3_OH/KOH are displayed in Figure 8. With increasing the scan rate, the reaction peaks became wider, and the oxidation peaks shifted towards more positive potentials. In similar way, the reverse scan’s reduction peaks shifted towards more negative potentials. The current densities increased with increasing the scan rate, which indicated that the reaction process was kinetically limited and controlled by diffusion. For each tested electrode demonstrated a linear correlation between the current density and the square root of the scan rate (Figure 8) which indicated that the methanol oxidation reaction was controlled by the diffusion process [55].

**Figure 9:**
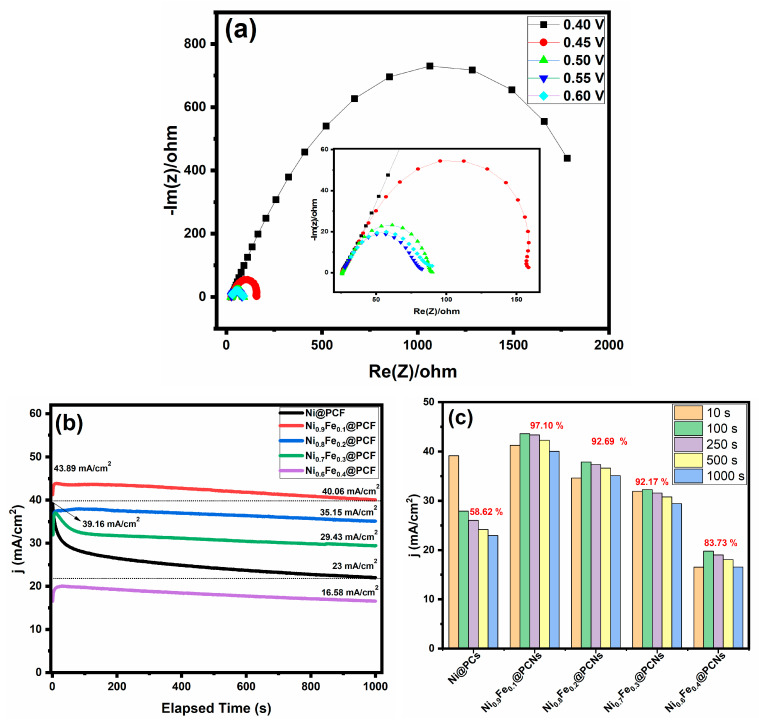
(**a**) Nyquist plots of Ni_0.9_Fe_0.1_@PCNs at various overpotentials in 1 M KOH + 2 M MeOH; (**b**) chronoamperometry at a potential of 0.5 V in 2.0 M methanol; (**c**) activity retention of catalyst samples.

To gain a better understanding of the electrocatalytic activity of Ni_0.9_Fe_0.1_@PCNs, electrochemical impedance spectroscopy (EIS) measurements were performed in 1 M KOH + 2 M methanol at various overpotentials, and the Nyquist plots of the EIS response are provided in Figure 9a. Similarly, the Nyquist plots show one semicircle after 0.4 V. The equivalent resistance was high before 0.4 V, indicating that the charge transfer was extremely weak. As the overpotential increased, the charge transfer resistance decreased. Chronoamperometry tests were performed at constant potential of 0.5 V in 1.0 M of KOH and methanol to assess the long-term activity and the stability of all electrodes for methanol oxidation for 1000 s, as shown in Figure 9b. In the first seconds, the current density of all of the iron-doped catalysts increased compared to the undoped catalysts (Ni@PCs). This result indicates the activation of the surface-active sites of the iron-doped catalysts in the first seconds. The current density over 1000 s for Ni_0.9_Fe_0.1_@PCNs was higher than that observed for the other catalysts. In contrast, the final current density of Ni@PCs was higher than that of Ni_0.6_Fe_0.4_@PCNs and lower than that of the other catalysts. However, the retention of the current density percentage after 1000 s for the iron-doped catalysts was higher than that for the undoped catalysts. The retention of the current density percentage of Ni_0.9_Fe_0.1_@PCNs, Ni_0.8_Fe_0.2_@PCNs, Ni_0.7_Fe_0.3_@PCNs, Ni_0.6_Fe_0.4_@PCNs, and Ni@PCs was 97.10, 92.69, 92.17, 83.73, and 58.62%, respectively, as shown in Figure 9c.

According to the obtained experimental results, the excellent electrocatalytic activity of the Ni_0.9_Fe_0.1_@PCNs towards MOR is attributed to several factors. The first factor is the synergistic effect between nickel and iron, which plays a remarkable role in enhancing the electrocatalytic activity and is superior to the monometallic-based catalyst. The second is that the distinct morphology and the porous structure of carbon nanosheets are inherently advantageous for boosting the electrocatalytic activity of NiFe sheets, because they allow for electrolyte diffusion into the catalyst structure, and good contact between the bimetallic sheets and the porous support facilitates the transfer of electrons. Furthermore, the preparation method is low cost and environmentally friendly, because it does not require the use of solvents, surfactants, or other agents; is completed in a single step; and the molten salt can be reused. The electrocatalytic activity of several of the bimetallic catalysts immobilized on various carbon materials, as well as the techniques utilized to prepare them, are summarized in Table 2. In alkaline media, it is clear that Ni_0.9_Fe_0.1_@PCNs catalyst is superior to those catalysts for methanol oxidation and has high stability.

## 3. Conclusions

In summary, this study developed a facile, environmentally friendly molten salt method for preparing porous carbon framework-supported nickel–iron nanoparticles (Ni-Fe@PCNs) without the use of organic solvents and surfactants. A series of electrocatalysts with different Ni/Fe ratios (Ni@PCs, Ni_0.9_Fe_0.1_@PCNs, Ni_0.8_Fe_0.2_@PCNs, Ni_0.7_Fe_0.3_@PCNs, and Ni_0.6_Fe_0.4_@PCNs) were prepared as anodic electrodes for methanol oxidation. The XRD analysis confirmed the formation of nickel–iron bimetallic particles with an fcc structure, and the average crystal size varied according to the ratio of iron, ranging between 15.5 and 30.6 nm. Microscopic studies confirmed the distribution of nickel–iron sheets on the porous carbon nanosheets. Electrochemical studies confirmed that the Ni_0.9_Fe_0.1_@PCN electrocatalyst had the highest ECSA, electrocatalytic activity, and stability for methanol oxidation, with a current density of 192 mA cm^–2^ and an activity retention of 97.1% after 1000 s. The Tafel slope value for the Ni_0.9_Fe_0.1_@PCNs (37.89 mV/dec) was lower than for the Ni@PCs (39.31 mV/dec) but higher when the iron content was higher than 10%. Despite the distinct morphology and excellent electrocatalytic activity of the catalyst prepared by molten salt, more research is required to understand the mechanism underlying the formation of metal/porous carbon nanosheets.

## 4. Materials and Methods

### 4.1. Materials and Synthesis Method

Iron acetate (FeAc, ≥99%), nickel acetate tetrahydrate (NiAc, ≥99.99), Nafion (5% wt./V), poly(vinyl alcohol) (PVA; 64,000 g/mol), lithium chloride (99%), potassium chloride (99.5%), methanol (99.9%), and potassium hydroxide (90%) were purchased from Sigma Aldrich (Munich, Germany).

### 4.2. Preparation Method

The catalysts were prepared using molten salt method as described by Yoon et al. with some modifications [33]. Initially, five samples of 10 g of KCl/LiCl were prepared as molten salt with a 55/45 *w*/*w* ratio, and then 0.5 g of NiAc and 0.5 g of PVA were added to each molten salt. After, different weight percentages of FeAc (0, 10, 20, 30, and 40% by the weight of NiAc) were weighed and then added to the previously molten salts. Each mixture was transferred to a blender and then ground for 15 min until a homogeneous powder was obtained. Then, each homogeneous mixture powder was transferred to a quartz crucible and placed in a tubular furnace for pyrolysis by heating at 750 °C for 3 h at a heating rate of 3 °C/min under nitrogen gas. After pyrolysis process was completed, the samples were cooled naturally to room temperature inside the tube furnace; then, the obtained mixture was ground and stirred in distilled water for two hours with stirring, filtered, and washed numerous times with distilled water to remove the molten salt. Finally, the samples were dried in an oven at 80 °C for 24 h.

**Figure 10:**
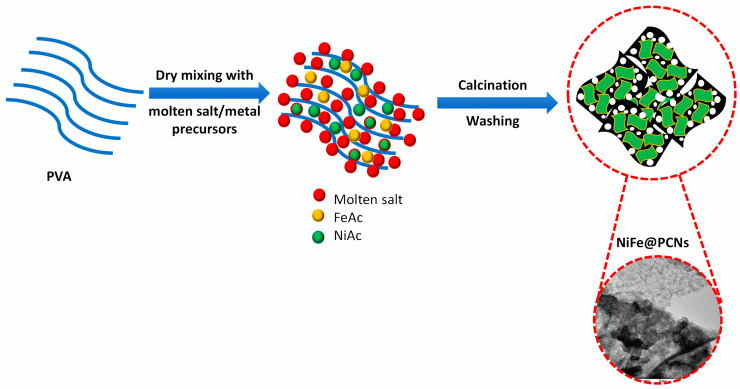
Schematic illustration of the NiFe@PCNs’ preparation.

Figure 10 displays a schematic illustration for the preparation method of NiFe@PCNs catalyst. Each prepared catalyst was defined based on the weight ratio of iron to nickel in the precursor and was defined as Ni_x_Fe_1−x_@PCNs (x = 1, 0.9, 0.8, 0.7, and 0.6).

### 4.3. Characterization

The morphology of the prepared catalysts was studied using a scanning electron microscope (SEM, Carl Zeiss, Dublin, CA, USA) and transmission electron microscope (TEM-1011, 100 Kv). To investigate the morphology of the samples by TEM, a drop of the dispersed solution (dispersed sample in methanol) was placed on a copper grid and evaporated into the air at room temperature.

The crystallinity and structure–composition phases were investigated by X-ray diffraction (XRD, BRUKER D8 ADVANCE). The measurement was performed at a scan rate of 7° in a *2θ* range between 5 and 80°.

The elemental composition and the oxidation states of the prepared catalysts were determined using X-ray photoelectron spectroscopy (XPS, ESCALAB 250Xi, Thermo Fischer Scientific, Waltham, MA USA). The powder sample was coated on carbon fiber paper with an area of 1 cm^2^, the irradiation region had a diameter of 100 m, and the residual pressure in the analysis chamber was less than 10^–7^ Torr.

The thermal decomposition of the used precursors for the catalysts’ preparation was studied by thermogravimetry/differential thermal analysis (TGA/TDG, TA, USA). A specific mass of the sample powder was weighed and then heated from 30 to 800 °C at a heating rate of 10 °C/min.

### 4.4. Electrochemical Measurements

The electrochemical performance of the prepared catalysts in alkaline media was investigated using a Versastat 3 potentiostat/galvanostat (VersaSTATE 3, AMETEK, Princeton, NJ, USA) by cyclic voltammetry (CV) and chronoamperometry (CA) techniques. The reference electrode is Ag/AgCl and the counter electrode is Pt wire. All powder samples were drop-casted onto a glassy carbon electrode (GC, 3 mm) before it was used as the working electrode. The catalyst inks were prepared by dispersing 0.002 g of the powder catalyst in 0.4 ml of isopropanol and 0.02 ml of Nafion solution (5.0 wt%) by ultrasonication for 30 min to form a suspension. Ten microliters of the catalyst ink were dropped onto the polished GC electrode and dried in the air overnight and under vacuum at 80 °C for 10 min. Before measuring the electrocatalytic activity of the catalyst towards the oxidation of methanol, an electrode was activated by performing 50 cycles in 1 M KOH. Its activity was then tested with three CVs at different concentrations of methanol, and the results from the third cycle are presented. The electrochemical impedance spectroscopy (EIS) experiments were performed with an AC amplitude of 10 mV throughout a frequency range of 10^−1^ to 10^5^ Hz.

## Figures and Tables

**Table 1:** XRD data for the catalysts and their crystallite size and carbon sheets number calculations.

Sample	2*θ*	FWHM	Size of Crystallite (D) (nm)	Average of D (nm)	d-Space (nm)	PCNs Number
Ni@PCs	44.40	0.2681	32.00	30.59	0.2038	
	51.76	0.2770	31.87		0.1765	
	76.31	0.3622	27.89		0.1247	
	26.05	1.02	8.007		0.3417	23.43
Ni_0.9_Fe_0.1_@PCNs	44.32	0.3007	28.52	27.73	0.2042	
	51.65	0.3186	27.70		0.1768	
	76.08	0.3740	26.97		0.1250	
	24.66	14.24	0.5711		0.3607	1.58
Ni_0.8_Fe_0.2_@PCNs	44.14	0.4019	21.33	21.69	0.20501	
	51.43	0.4078	21.62		0.1775	
	75.76	0.4548	22.13		0.1254	
	25.11	16.16	0.5035		0.3543	1.42
Ni_0.7_Fe_0.3_@PCNs	44.04	0.3653	23.46	23.55	0.2055	
	51.30	0.3769	23.38		0.1779	
	75.48	0.4221	23.80		0.1258	
	24.73	15.32	0.5307		0.3597	1.74
Ni_0.6_Fe_0.4_@PCNs	43.85	0.5289	16.19	15.51	0.2063	
	51.08	0.5473	16.09		0.1787	
	75.07	0.7033	14.24		0.1264	
	23.65	10.39	0.7810		0.3758	2.08

**Table 2:** Electrocatalytic performance and durability of the Ni0.9Fe0.1@PCNs compared with previously reported electrocatalysts.

Electrocatalyst	Synthesis Method	j (mA/cm^2^)	Catalytic Activity Retention (%)	Ref.
NiCo_2_O_4_/Ni foam	Microwave-assisted synthesis	10	91.7% at 1000 s	[56]
Ni_0.75_Cu_0.25_	Electrodeposition	84	~91% at 1200 s	[57]
Ni_0.2_Co_0.2_/Gr	Impregnation/calcination	75	-	[58]
Cu/NiCu nanowires	Wet synthesis	34.9	~95% at 10,000 s	[59]
Co/NCNFs/graphite	Electrospinning/calcination	90	~80% at 1000 s	[60]
NiCo/N-doped graphene	Electrodeposition	88.04	~70% at 2000 s	[61]
NiCo_2_O_4_/rGO	Hydrothermal/calcination	78	~19.2% at 3000 s	[62]
NiCo/NiO-CoO	Hydrothermal/carbonization	178	~38% at 3570s	[63]
NiSn NPs	Coreduction	50	~79% at 5000 s	[64]
Co-Cu/CNFs	Electrospinning/calcination	17	~70% at 900 s	[65]
Ni_0.9_Fe_0.1_@PCNs	Molten salt	191.3	97.1% at 1000 s	This study

## Data Availability

The data presented in this study are available on request from the corresponding author.
